# Improving Processing and Performance of Pure Lignin Carbon Fibers through Hardwood and Herbaceous Lignin Blends

**DOI:** 10.3390/ijms18071410

**Published:** 2017-07-01

**Authors:** Omid Hosseinaei, David P. Harper, Joseph J. Bozell, Timothy G. Rials

**Affiliations:** Center for Renewable Carbon, University of Tennessee, 2506 Jacob Drive, Knoxville, TN 37996, USA; ohossein@utk.edu (O.H.); jbozell@utk.edu (J.J.B.); trials@utk.edu (T.G.R.)

**Keywords:** lignin, carbon fiber, hardwood, herbaceous, nuclear magnetic resonance (NMR) spectroscopy, thermal properties, thermostabilization, tensile properties

## Abstract

Lignin/lignin blends were used to improve fiber spinning, stabilization rates, and properties of lignin-based carbon fibers. Organosolv lignin from Alamo switchgrass (*Panicum virgatum*) and yellow poplar (*Liriodendron tulipifera*) were used as blends for making lignin-based carbon fibers. Different ratios of yellow poplar:switchgrass lignin blends were prepared (50:50, 75:25, and 85:15 *w/w*). Chemical composition and thermal properties of lignin samples were determined. Thermal properties of lignins were analyzed using thermogravimetric analysis and differential scanning calorimetry. Thermal analysis confirmed switchgrass and yellow poplar lignin form miscible blends, as a single glass transition was observed. Lignin fibers were produced via melt-spinning by twin-screw extrusion. Lignin fibers were thermostabilized at different rates and subsequently carbonized. Spinnability of switchgrass lignin markedly improved by blending with yellow poplar lignin. On the other hand, switchgrass lignin significantly improved thermostabilization performance of yellow poplar fibers, preventing fusion of fibers during fast stabilization and improving mechanical properties of fibers. These results suggest a route towards a 100% renewable carbon fiber with significant decrease in production time and improved mechanical performance.

## 1. Introduction

Lignin has been explored as a precursor for low-cost and bio-derived carbon fibers since the 1960s [1,2,3], but it has gained significant attention recently as many industries seek to find renewable replacements for petroleum derived carbon [4,5]. Different methods of spinning, including wet and dry spinning, melt-spinning, and electrospinning, have been investigated to manufacture lignin-based carbon fibers [1,2,4,5,6,7,8,9,10,11]. The most dominant and preferred method, especially for producing low cost fibers for composite and structural applications, is melt-spinning [2,4,12]. Melt-spinning requires lignin to be fusible (an ability to melt and flow), without undergoing extensive thermal-induced depolymerization and/or condensation reactions during extrusion. Depolymerization produces volatiles that lead to defects (mainly pores) on the surface of fibers, while condensation restricts lignin’s thermal mobility and melt flow characteristics, consequently affecting spinning performance.

Manufacturing lignin carbon fibers involves multiple processing steps of melt-spinning, oxidative thermostabilization, and carbonization. Different lignins, depending on their botanical source and extraction process, have different characteristics and behave differently during melt-spinning and conversion to carbon fiber [2,3,13]. Hardwood lignin, especially organosolv, has demonstrated greater thermal mobility and better spinnability, while lignins from softwoods and grasses have better performance during thermostabilization and conversion processes [2,3,6,14,15]. Lignins with a high number of guaiacyl (G) units (softwoods and grasses) have a more condensed and cross-linked structure, which limits thermal mobility and causes difficulty in melt-spinning [2,3,15]. In the case of organosolv hardwood lignin, ethoxylation of its side chain is responsible for its high thermal mobility and great spinnability [3,16]. On the other hand, the more condensed structure (C–C linkages) and unoccupied C_5_ sites in high G content lignins (grasses and softwood) promotes faster thermostabilization and reduces carbon fiber production time [3,15].

Increasing the thermal mobility of lignin to improve melt-spinnability has been the focus of much previous work, especially in the case of kraft lignin. This includes plasticizing lignin through blending with synthetic polymers such as polyethylene (PE) [17], polyethylene oxide (PEO) [2,17,18], polyethylene terephthalate (PET) [17,18,19], polypropylene (PP) [17,18,19,20,21], and polyvinyl alcohol (PVA) [18,22]. Thunga et al. [11] used a blend of chemically modified softwood kraft lignin (butyrated) and polylactic acid (PLA) for melt-spinning. In addition, chemically modified lignins, such as acetylated [23], hydrogenated [24], and phenolated [25] lignins also have been tried to improve melt-spinnability. Chemical modification is costly and in some cases, such as acetylation, produce porous fibers [23,26]. Purification and fractionation, through ultrafiltration [14], pH-fractionation [27], and solvent extraction [6], are other methods which have been used for obtaining lignin with high thermal mobility and improved melt-spinning performance.

In principle, blending lignin with other polymers should be an easy and low-cost method to improve spinnability of lignin. However, in the case of polymer blends, miscibility is an important factor that is impacted by hydrogen bonding and acid-base interaction between components of the blend [18]. For example, it has been shown that most polymers such as PP, PVA, and PE are immiscible with lignin [17]. These immiscible blends can negatively affect melt-spinnability and degrade fiber processing in lignins such as Alcell or hardwood kraft, or result in the formation of hollow and porous carbon fiber [17,19,20]. Both PEO and PET can form a miscible blend with lignin, especially PEO, which forms a strong intermolecular hydrogen bonding network [16,18]. However, there are limitations, such as difficulty in thermostabilization of PEO/lignin blends and the high melting temperature of PET, which is above the thermal decomposition point for lignin [2,19].

Lignin/lignin blends, plasticizing an infusible lignin with a fusible lignin, could be an approach to improve melt processing while preventing problems related to the miscibility of lignin and synthetic polymers. Nordstrom et al. [14] were able to melt-spin softwood kraft lignin, which is originally infusible, by plasticizing it with membrane fractionated hardwood kraft lignin. In our previous work, we demonstrated how thermal properties and chemical structure of organosolv hardwood and herbaceous lignins results in differences in melt-spinning, thermostabilization performance, and properties of resulting carbon fibers [3]. Organosolv hardwood lignin, due to its lower number of aliphatic hydroxyl groups and phenolic acids, presented better spinnability and produced stronger fibers with fewer defects. On the other hand, switchgrass lignins were difficult to process into fibers and produced fibers with defects and lower tensile properties. However, the higher number of G units and more condensed structure in switchgrass lignin resulted in faster thermostabilization [3]. The goal of this paper is to use different ratios of organosolv switchgrass and yellow poplar lignin for producing melt-spun lignin-based carbon fibers. Organosolv hardwood lignin (yellow poplar) will plasticize and improve spinning of switchgrass lignin, while switchgrass lignin will help to increase the thermostabilization rate and decrease the processing time to convert lignin to carbon fiber.

## 2. Results and Discussion

### 2.1. Properties of Lignins

We extensively evaluated physical and chemical properties of switchgrass and yellow poplar organosolv lignins from the same process in our previous study [3]. Here, we briefly discuss some important physical/chemical properties of lignin. Purity, acid insoluble and acid soluble lignin content, and elemental composition of lignin samples are summarized in Table 1. Both lignins have low amounts of impurities, which is desirable for making carbon fibers (Table 1). Similar to observations in our previous paper, switchgrass lignin has a higher amount of impurities which include residual hemicelluloses, ash, and protein-based components (as indicated by its higher nitrogen content). Impurities can prevent fusion and flow during melt-spinning, cause defects on the surface of carbon fibers, and decrease fiber carbon yield [2,3].

**Figure 1:**
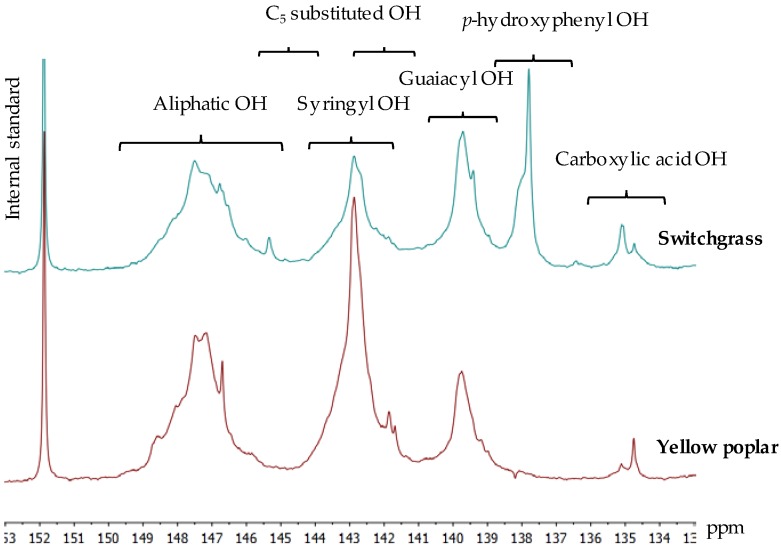
Quantitative ^31^P NMR spectra and signal assignment of lignin samples.

Hydroxyl groups are important functional groups in lignin that affect its physical properties, especially *T_g_*, and consequently its melt and flow behavior [3,16,28]. Based on the ^31^P NMR (phosphorus-31 nuclear magnetic resonance) results, yellow poplar lignin has a higher number of phenolic hydroxyl groups while switchgrass lignin has a higher number of aliphatic hydroxyl groups (Figure 1; Table 2). Aliphatic hydroxyl groups result in the formation of intermolecular hydrogen bonds, which decrease molecular motion, increase *T_g_*, and limit fusibility [16,28]. In addition, switchgrass lignin possesses a significant number of *p*-hydroxyphenyl hydroxyl groups, derived from *p*-hydroxyphenyl (H) units and *p*-coumarates (Table 2), which also can negatively affect melt-spinning performance [3]. The combined presence of aliphatic hydroxyl groups and phenolic acids, such as *p*-coumarates in switchgrass lignin, makes continuous spinning very difficult by increasing its melt viscosity and reducing its thermal stability [3].

According to differential scanning calorimeter (DSC) results, *T_g_* of switchgrass lignin was higher than yellow poplar lignin (Table 3). Higher *T_g_* indicates less thermal mobility and restriction in molecular motion, which also will affect fusibility of lignin. This difference in the *T_g_* of the lignins could be due to multiple factors such as the higher content of impurities, and a higher number of aliphatic hydroxyl groups in switchgrass lignin. On the other hand, lignin with a higher *T_g_* can potentially thermostabilize faster and have shorter processing time compared to lignin with a lower *T_g_* [4]. *T_g_* also affects thermal softening and the melt flow temperature of lignin, as presented in Table 3; switchgrass lignin has a higher melt temperature.

**Figure 2:**
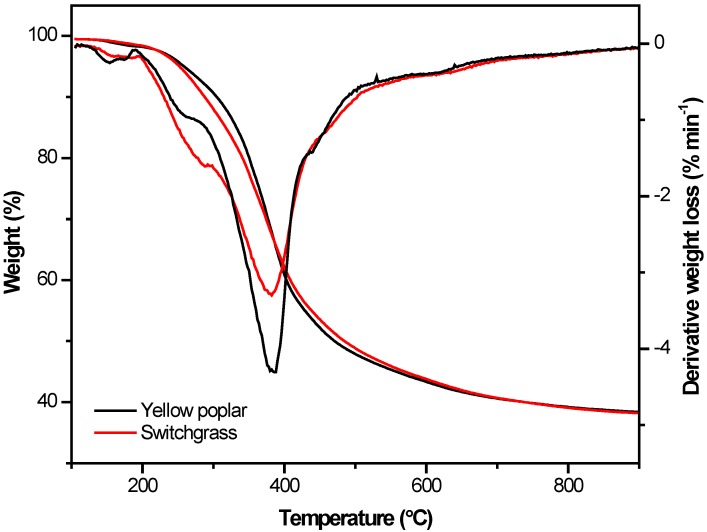
Thermal decomposition of switchgrass and yellow poplar lignins.

The thermal decomposition profile of lignins shows a clear difference, as switchgrass lignin has lower thermal stability in the 200–300 °C range (Figure 2; Table 3). The mass loss in this range is due to thermal decomposition of aliphatic groups, phenolic acids, and residual hemicellulose [3]. Carbon fibers made from switchgrass lignin have more defects and pores caused by the presence of less thermally stable and volatile materials [3]. The combination of higher processing temperatures and lower thermal stability in this processing range makes producing pure switchgrass lignin fibers of high quality nearly impossible without modification.

### 2.2. Fiber Spinning and Conversion to Carbon Fiber

**Figure 3:**
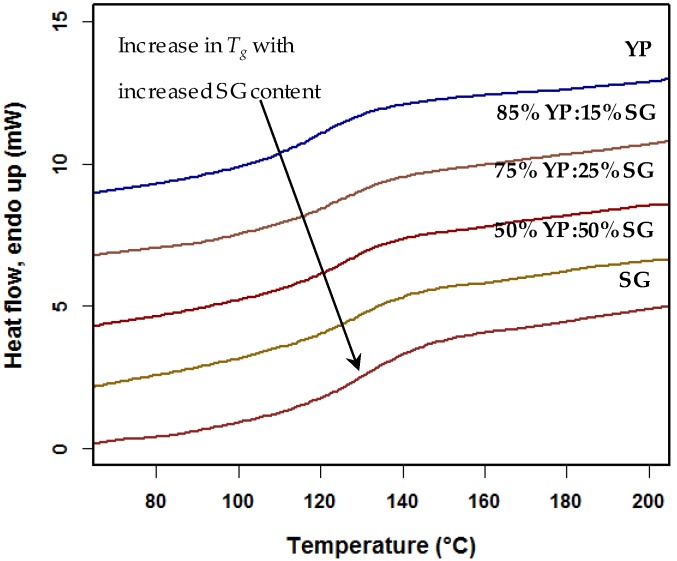
Differential scanning calorimeter (DSC) thermograms of switchgrass, yellow poplar, and vary ratios (YP:SG *w/w*) lignin composition.

The *T_g_* values of different blend ratios stand between *T_g_* of pure switchgrass and yellow poplar lignin. As expected, samples with 50% switchgrass lignin have the highest *T_g_* value (Table 4). DSC curves of all blend samples have a single transition, which indicates a miscible blend (Figure 3) [14,19]. Immiscible lignin/polymer blends, such as lignin/PP, present two separate *T_g_* values for the individual polymers [19]. Immiscible blends also exhibit phase separation, fiber breakage, or a twisted fiber geometry during extrusion or subsequent thermal processing.

All blends showed good spinnability and the ability to be continuously spun. Continuous melt spinning of pure switchgrass lignin was not possible, as it possessed a relatively high melt viscosity and presence of volatiles [3]. However, blending switchgrass with yellow poplar lignin remarkably improved spinning performance of switchgrass lignin, even for blends with high switchgrass ratio (50%).

Although there was a slight difference in *T_g_* and melt temperature of individual lignin samples, extrusion was performed at the same temperature (180 °C) for all ratios. This temperature is relatively low and far below the thermal decomposition temperature of lignins (252–259 °C). Keeping the extrusion temperature as low as possible prevents the formation of volatiles and helps produce fibers with fewer defects [14]. Yellow poplar lignin helps to decrease the melt viscosity of switchgrass lignin while making it possible to extrude at a lower temperature, decreasing the formation of volatiles. Previously, we reported non-continuous extrusion of neat switchgrass lignin at a temperature of 190 °C [3]. In this work, continuous spinning of the blend at 180 °C was possible. The winding speed also significantly improved. Fibers from blends were spun at about 60 m min^−1^, while neat switchgrass lignin could only be spun at 18 m min^−1^ [3].

### 2.3. Properties of Lignin-Based Carbon Fibers

**Figure 4:**
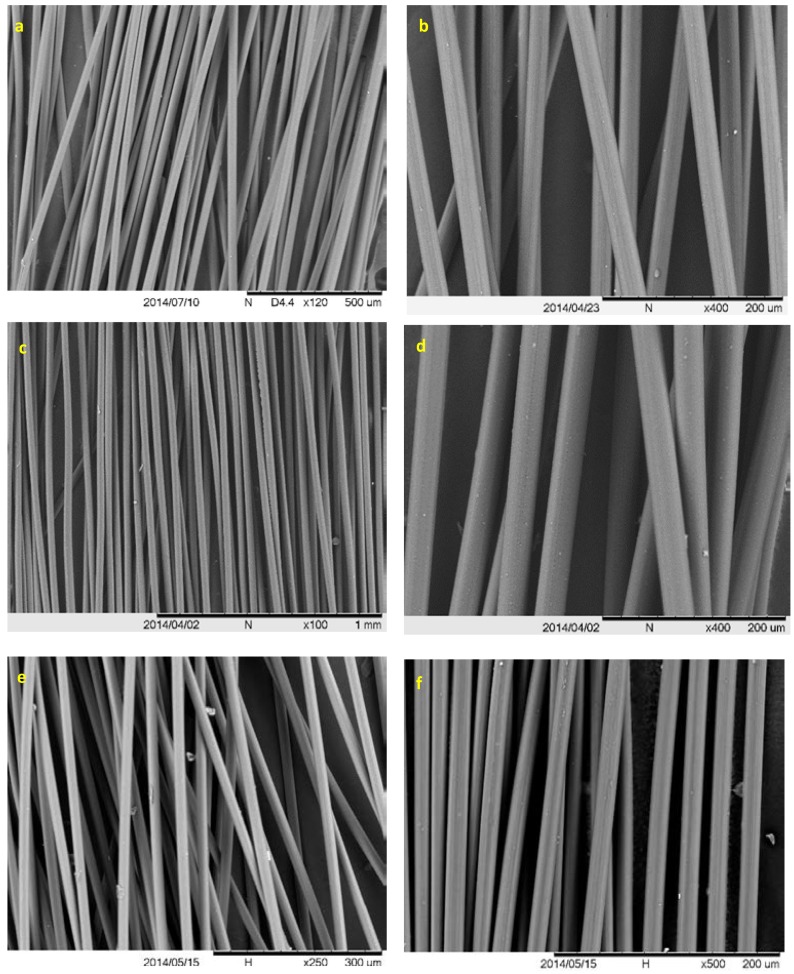
SEM images of carbon fibers made from switchgrass and yellow poplar lignin blends: (**a**,**b**) 50% YP:50% SG thermostabilized at heating rate of 0.05 °C min^−1^, (**c**,**d**) 50% YP:50% SG thermostabilized at heating rate of 0.5 °C min^−1^, (**e**) 75% YP:25% SG thermostabilized at heating rate of 0.05 °C min^−1^, and (**f**) 85% YP:15% SG thermostabilized at heating rate of 0.05 °C min^−1^.

Prior to carbonization, lignin fibers were oxidatively thermostabilized at different rates to evaluate the effect of blending on thermostabilization performance (Table 5). The thermostabilization rate needs to be slow enough to prevent the fusion of fibers. Lower S/G ratios and a more condensed structure of switchgrass lignin (higher number of C–C linkages) facilitates faster stabilization [3]. The 50% blend had the best stabilization performance and did not fuse even at the fastest rate (Table 5; Figure 4). Increasing the ratio of yellow poplar lignin resulted in slightly decreasing stabilization rate performance and resulted in some fiber fusion, especially in the blend with the lowest level of switchgrass lignin (Table 5). Unlike previously reported results for melt spinning of neat switchgrass lignin [3], pores or surface defects were not observed on the fibers (Figure 4). The thermostabilization performance of blended fibers was even better than previously reported data for neat samples from both biomass sources [3]. In the case of yellow poplar lignin, the improvement is directly related to the effect of switchgrass lignin, which facilitates crosslinking and condensation reactions during thermostabilization. The higher *T_g_* of switchgrass lignin in this paper compared to our previous report is the reason that fibers made from a blend of this lignin can be thermostabilized faster compared to fibers prepared from neat switchgrass lignin [3]. Higher *T_g_* facilitates thermostabilization and makes it possible to decrease the processing time required for manufacturing carbon fiber [4].

**Figure 5:**
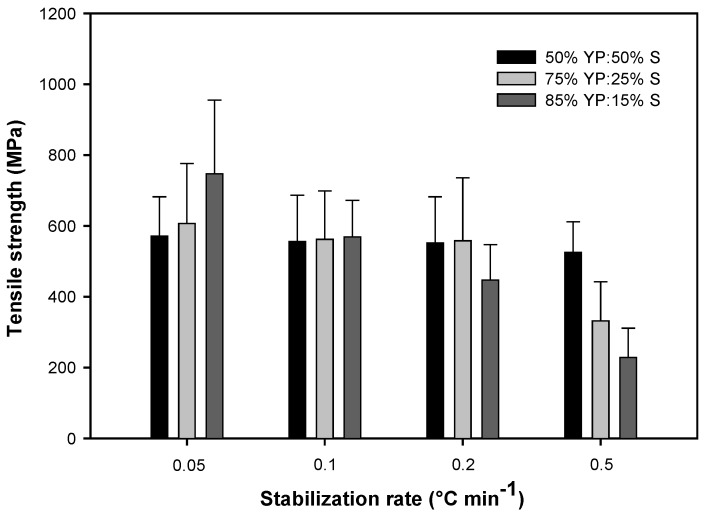
Effect of thermostabilization rate on tensile strength of carbon fibers made from different ratios of switchgrass and yellow polar lignin blends.

**Figure 6:**
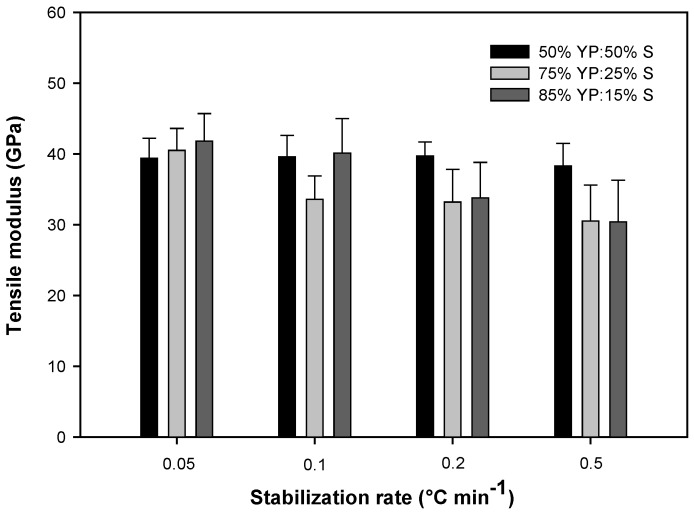
Effect of thermostabilization rate on tensile modulus of carbon fibers made from different ratios of switchgrass and yellow polar lignin blends.

The mechanical properties of carbon fibers are summarized in Table 6 and Figure 5 and Figure 6. The data were analyzed with analysis of variance (ANOVA) followed by Tukey test for comparing means using JMP software (Supplementary Materials, Table S1). In all blends, the diameter of the fibers increased slightly at increased stabilization rates (Table 6). It has been shown that slower stabilization rates induce oxidation, increase mass loss, and result in decreased fiber diameters [3,29]. Therefore, faster stabilization rates can increase the final carbon fiber yield. Fibers with the lowest switchgrass lignin content (15%) have the smallest diameter, which indicates switchgrass lignin increases extensional viscosity and prevents stretching of the fibers during spinning. These observations are consistent with results previously reported about the effect of botanical lignin source on stabilization rate and properties of lignin-based carbon fibers [3].

The tensile strength of fibers at all blend ratios decreased slightly by increasing the stabilization rate (Table 6). The samples with the highest switchgrass content (50%) showed no statistically significant changes in tensile properties based on different stabilization rates (Table 6; Supplementary Materials, Table S1). This could be mainly based on stabilization performance of fibers, since the sample that had the best stabilization performance had no fusion observed, even at the highest stabilization rate. Fibers with the lowest content of switchgrass lignin (15%) and the lowest stabilization rate (0.05 °C min^−1^) had the best tensile strength, which was significantly different from other samples (Table 6; Supplementary Materials, Table S1). Switchgrass lignin, compared to yellow poplar lignin, can produce more volatiles and form pores or defects on the surface of fibers. This decreases the mechanical properties of carbon fibers [3]. Therefore, fibers with high switchgrass lignin content (50%) had the lowest tensile strength at the same stabilization rate (0.05 °C min^−1^). Based on these results, the sample with the lowest ratio of switchgrass lignin and which stabilized at the slowest rate (0.05 °C min^−1^), to prevent fusion, had better tensile strength compared to the samples with high switchgrass lignin content. On the other hand, at the fastest stabilization rate (0. 5 °C min^−1^), the samples with high switchgrass lignin content (50%), which did not fuse, had the highest tensile strength (Table 6, Figure 5 and Figure 6).

Tensile modulus of carbon fibers also followed the same trend as tensile strength, where the blend of 50% switchgrass lignin had the best performance in different stabilization rates, and the sample with 15% switchgrass lignin had the highest tensile modulus at the slowest stabilization rate (Table 6). However, based on statistical analysis, the differences between tensile modulus of all blend ratios stabilized at a rate of 0.05 °C min^−1^ were not significant (Supplementary Materials, Table S1). For both tensile strength and tensile modulus, the lowest values were observed in samples with 15% and 25% switchgrass lignin when stabilized at the fastest stabilization rate (0.5 °C min^−1^). These values were significantly different from other values (Table 6; Supplementary Materials, Table S1). The highest tensile strength was observed in the sample with 15% switchgrass lignin when stabilized at the slowest stabilization rate (0.05 °C min^−1^). This value was significantly different from other values (Table 6; Supplementary Materials, Table S1). Based on the results, increasing switchgrass lignin content can accelerate stabilization and prevent fusion, but due to its effects on formation of defects, it can reduce tensile strength when used at high quantity.

## 3. Materials and Methods

### 3.1. Materials

Organosolv lignins were obtained from Alamo switchgrass (knife milled to 2–5 cm) and pulp grade yellow poplar chips (about 4 cm^2^ and thicknesses of 0.5–1 cm) using a previously described organosolv fraction method [3,30,31]. In brief, organosolv fractionation was performed in a flow-through reactor with a 16:34:50 *w/w* mixture of methyl isobutyl ketone (MIBK), ethanol, and water in the presence of sulfuric acid (0.05 M) at a temperature of 160 °C for 120 min (severity factor of 2.50). The collected black liquor separated to a lignin-rich solvent phase and a hemicellulose-rich aqueous phase by adding NaCl (10 g per 100 mL of deionized water in the initial solvent mixture) in a separatory funnel. Lignin from both phases was isolated by rotary evaporation of each phase until all solvent was removed. The isolation process continued by trituration of the solid residue with diethyl ether and final washing with deionized water to remove possible impurities. The final lignin was filtered through a paper filter and dried in a vacuum oven at 80 °C for 12 h.

### 3.2. Chemical and Elemental Composition of Lignin Samples

The ash content and lignin content (acid soluble plus acid insoluble lignin) of the lignin samples was determined using standard procedures [32,33]. Elemental compositions of lignin samples (carbon, hydrogen, and nitrogen content) were measured using a PerkinElmer 2400II CHNS/O combustion elemental analyzer. These measurements were performed in triplicate.

### 3.3. Thermal Properties of Lignin Samples

The lignin zero shear melt flow temperature (*T_m_*) was determined optically using a Fisher-Johns melting point apparatus. Thermal decomposition of lignin samples was studied using a PerkinElmer Pyris 1 thermogravimetric analyzer. Thermogravimetric analyses (TGA) were conducted in duplicate using approximately 5–7 mg specimens that were heated from room temperature to 105 °C at a heating rate of 10 °C min^−1^, held at this temperature for 10 min to remove moisture, and then heated to 950 °C at the same heating rate under a nitrogen atmosphere (10 mL min^−1^). Glass transition temperatures (*T_g_*) were determined in triplicate on approximately 2 mg samples of lignin using a PerkinElmer Diamond differential scanning calorimeter (DSC) calibrated with indium standard at 100 °C min^−1^. Each specimen was heated from 25 °C at a rate of 100 °C min^−1^ under nitrogen (UHP, 20 mL min^−1^) to 200 °C and held at that temperature until the change in thermal energy from the sample was zero to expel any remaining moisture in the sample and erase the thermal history of the sample. The sample was then cooled to 25 °C at a rate of 100 °C min^−1^ and again heated to 220 °C at the same heating rate. The second trace was used for the calculation of *T_g_* and the corresponding heat capacity (∆*C*_p_).

### 3.4. ^31^P NMR Spectroscopy of Lignin Samples

Hydroxyl groups of lignin samples were measured using quantitative ^31^P NMR spectroscopy after derivatization of lignin with 100 µL of 2-chloro-4,4,5,5-tetramethyl-1,3,2-dioxaphospholane (TMDP) [34,35]. The derivatized samples (30 mg) were dissolved in 0.75 mL of pyridine and deuterated chloroform (1.6:1 *v/v*) and mixed with 100 μL of a solution of *N*-hydroxy-5-norbornene-2,3-dicarboxylic acid imide (10 mg mL^−1^) and chromium(III) acetylacetonate (5 mg mL^−1^) as internal standard and relaxation agent, respectively. ^31^P NMR spectra were acquired using an inverse-gated decoupling pulse sequence with a 90° pulse angle, 25 s relaxation delay, and 256 scans.

### 3.5. Spinning and Conversion to Carbon Fiber

Yellow poplar and switchgrass lignins were mixed at ratios of 50:50, 75:25, and 85:15 (*w/w*) and the blend used for melt spinning. Melt spinning of the lignins was performed using a Haake MiniLab counter-rotating twin-screw extruder (Thermo Scientific), equipped with a 200 µm custom-built die. The temperature of the extruder and spinneret were 180 and 185 °C, respectively. A rotating cylinder (76 mm diameter), rotating at 250 rpm, was used to collect fibers.

Lignin fibers were oxidatively thermostabilized by heating the samples to 250 °C under air at selected rates (0.05, 0.1, 0.2, and 0.5 °C min^−1^) and then holding the samples for 30 min at 250 °C in a Heratherm OGH60 oven (Thermo Scientific). The stabilized fibers were carbonized in a Lindberg/Blue M 25 mm tube furnace (Thermo Scientific) by heating from room temperature to 600 °C at a rate of 3 °C min^−1^, holding for 5 min at 600 °C, heating from 600 to 1000 °C at a rate of 5 °C min^−1^ and holding for 15 min at 1000 °C, under a nitrogen flow of 0.2 L min^−1^. The tensile properties of carbon fibers were measured according to the ASTM standard (ASTM C1557-03) using an Instron 5943 single column tabletop testing system [36]. Results were an average of 40 fibers per each sample.

## 4. Conclusions

Yellow poplar and switchgrass lignins produce miscible blends that do not phase separate with thermal processing. Yellow poplar lignin improves the spinning performance of switchgrass blends. Higher switchgrass content enables thermostabilization of fibers at high rates without fusing. The fibers with the highest switchgrass lignin content (50%) did not show any fusion, even at the fastest stabilization rate (0.5 °C min^−1^). Mechanical properties of carbon fibers were highest in the samples with the lowest switchgrass lignin content and stabilized at the lowest rate. However, samples with the highest switchgrass lignin content had consistent mechanical properties across all stabilization rates evaluated. This study provides a rational approach for further development of carbon fibers from lignin by combining the best performance properties of each lignin to improve overall fiber process and performance.

## Figures and Tables

**Table 1:** The composition of switchgrass and yellow poplar lignin samples.

Parameter	Switchgrass	Yellow Poplar
Purity (%) ^1^	93.2	96.2
Ash (%)	0.30	0.17
C (%)	64.4	64.5
H (%)	5.70	5.89
N (%)	0.78	0.26
O (%) ^2^	28.8	29.2

^1^ Purity was sum of acid soluble and acid insoluble lignin. ^2^ Subtracted from C, H, N, and ash.

**Table 2:** Hydroxyl group contents of lignin samples obtained by quantitative ^31^P NMR spectroscopy (mmol g^−1^).

Sample ID	Carboxylic Acid OH (COOH)	Phenolic OH				Total Phenolic OH	Aliphatic OH
		*p*-Hydroxyphenyl	Condensed Phenolic	Guaiacyl	Syringyl		
Yellow poplar	0.05	0.00	0.35	0.72	2.41	3.48	1.58
Switchgrass	0.11	0.52	0.40	0.74	0.69	2.35	1.88

**Table 3:** Summary of key thermal properties of switchgrass and yellow poplar lignin samples.

Parameter	Yellow Poplar	Switchgrass
*T_g_* (°C)	119	129
Delta C_p_ (J g^−1^°C^−1^)	0.43	0.31
*T_m_* (°C)	150	160
*T_d_* (°C)	259	252
DTG peak temperature (°C)	382	378
DTG Peak value (% min^−1^)	−4.27	−3.27
Mass at 300 °C (%)	90.6	88.0
Mass at 400 °C (%)	60.9	62.1
Mass at 500 °C (%)	47.9	48.8
Residual char (%)	38.3	38.1

*T_g_*: Glass transition temperature; *T_d_*: thermal decomposition temperature (5% weight loss temperature); and *T_m_*: zero shear melt point temperature; DTG: derivative thermogravimetric analysis.

**Table 4:** *T_g_* of switchgrass (SG) and yellow polar (YP) lignin blends (*w/w*).

Parameter	50% YP:50% SG	75% YP:25% SG	85% YP:15% SG
*T_g_* (°C)	127	125	122
Delta Cp (J g^−1^ °C^−1^)	0.35	0.37	0.41

**Table 5:** Thermostabilization behavior of lignin fibers made from different ratios of switchgrass and yellow polar lignin blends.

Source	Heating Rate (°C min^−1^)			
	0.05	0.1	0.2	0.5
50% YP:50% SG	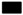	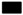	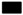	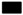
75% YP:25% SG	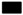	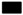	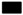	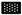
85% YP:15% SG	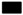	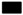	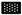	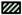

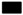
 Excellent (without fusion and sticking together); 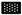 Good (slightly stick together);and 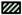 Moderately fused.

**Table 6:** Mechanical properties of carbon fibers made from different ratios of switchgrass and yellow poplar lignin blend ^a^.

Source	Stabilization Rate (°C min^−1^)	Diameter (µm)	Tensile Strength (MPa)	Tensile Modulus (GPa)	Strain at Break (%)
50% YP:50% SG	0.05	24.1 (2.1)	571 (111)	39.4 (2.8)	1.07 (0.16)
50% YP:50% SG	0.1	26.8 (1.2)	556 (131)	39.6 (3.0)	1.23 (0.24)
50% YP:50% SG	0.2	29.3 (1.4)	552 (130)	39.7 (2.0)	1.19 (0.25)
50% YP:50% SG	0.5	32.0 (1.4)	525 (87)	38.3 (3.2)	1.25 (0.18)
75% YP:25% SG	0.05	18.8 (1.3)	607 (169)	40.5 (3.1)	1.11 (0.30)
75% YP:25% SG	0.1	21.4 (3.3)	562 (137)	33.6 (3.3)	1.32 (0.13)
75% YP:25% SG	0.2	22.9 (2.0)	558 (178)	33.2 (4.6)	1.41 (0.26)
75% YP:25% SG	0.5	31.7 (3.4)	332 (110)	30.5 (5.1)	1.36 (0.35)
85% YP:15% SG	0.05	15.7 (1.1)	747 (208)	41.8 (3.9)	1.18 (0.40)
85% YP:15% SG	0.1	17.0 (1.0)	569 (103)	40.1 (4.9)	1.09 (0.27)
85% YP:15% SG	0.2	19.1 (1.6)	447 (100)	33.8 (5.0)	1.35 (0.29)
85% YP:15% SG	0.5	23.4 (1.9)	229 (82)	30.4 (5.9)	1.15 (0.35)

^a^ Standard deviations are shown in parentheses.

## Supplementary Materials

Supplementary materials can be found at www.mdpi.com/1422-0067/18/7/1410/s1.

## References

[1] Otani S., Fukuoka Y., Igarashi B., Sasaki K. (1969). Method For Producing Carbonized Lignin Fiber. U.S. Patent.

[2] Kadla J.F., Kubo S., Venditti R.A., Gilbert R.D., Compere A.L., Griffith W. (2002). Lignin-based carbon fibers for composite fiber applications. Carbon.

[3] Hosseinaei O., Harper D.P., Bozell J.J., Rials T.G. (2016). Role of physicochemical structure of organosolv hardwood and herbaceous lignins on carbon fiber performance. ACS Sustain. Chem. Eng..

[4] Baker D.A., Rials T.G. (2013). Recent advances in low-cost carbon fiber manufacture from lignin. J. Appl. Polym. Sci..

[5] Frank E., Steudle L.M., Ingildeev D., Spörl J.M., Buchmeiser M.R. (2014). Carbon fibers: Precursor systems, processing, structure, and properties. Angew. Chem. Int. Ed..

[6] Baker D.A., Gallego N.C., Baker F.S. (2012). On the characterization and spinning of an organic-purified lignin toward the manufacture of low-cost carbon fiber. J. Appl. Polym. Sci..

[7] Dallmeyer I., Ko F., Kadla J.F. (2010). Electrospinning of technical lignins for the production of fibrous networks. J. Wood Chem. Technol..

[8] Ruiz-Rosas R., Bedia J., Lallave M., Loscertales I.G., Barrero A., Rodríguez-Mirasol J., Cordero T. (2010). The production of submicron diameter carbon fibers by the electrospinning of lignin. Carbon.

[9] Baker D.A., Hosseinaei O. (2014). High glass transition lignins and lignin derivatives for the manufacture of carbon and graphite fibers. U.S. Patent.

[10] Zhang M., Ogale A.A. (2014). Carbon fibers from dry-spinning of acetylated softwood kraft lignin. Carbon.

[11] Thunga M., Chen K., Grewell D., Kessler M.R. (2014). Bio-renewable precursor fibers from lignin/polylactide blends for conversion to carbon fibers. Carbon.

[12] Meek N., Penumadu D., Hosseinaei O., Harper D., Young S., Rials T. (2016). Synthesis and characterization of lignin carbon fiber and composites. Compos. Sci. Technol..

[13] Sammons R.J., Harper D.P., Labbé N., Bozell J.J., Elder T., Rials T.G. (2013). Characterization of organosolv lignins using thermal and FT-IR spectroscopic analysis. BioResources.

[14] Nordström Y., Norberg I., Sjöholm E., Drougge R. (2013). A new softening agent for melt spinning of softwood kraft lignin. J. Appl. Polym. Sci..

[15] Norberg I., Nordström Y., Drougge R., Gellerstedt G., Sjöholm E. (2012). A new method for stabilizing softwood kraft lignin fibers for carbon fiber production. J. Appl. Polym. Sci..

[16] Kubo S., Kadla J.F. (2004). Poly(ethylene oxide)/organosolv lignin blends: Relationship between thermal properties, chemical structure, and blend behavior. Macromolecules.

[17] Kadla J.F., Kubo S., Gilbert R.D., Venditti R.A., Hu T.Q. (2002). Lignin-based carbon fibers. Chemical Modification, Properties, and Usage of Lignin.

[18] Kadla J.F., Kubo S. (2004). Lignin-based polymer blends: Analysis of intermolecular interactions in lignin–synthetic polymer blends. Compos. Part A.

[19] Kubo S., Kadla J.F. (2005). Lignin-based carbon fibers: Effect of synthetic polymer blending on fiber properties. J. Polym. Environ..

[20] Kubo S., Yoshida T., Kadla J.F. (2007). Surface porosity of lignin/pp blend carbon fibers. J. Wood Chem. Technol..

[21] Kadla J.F., Kubo S., Venditti R.A., Gilbert R.D. (2002). Novel hollow core fibers prepared from lignin polypropylene blends. J. Appl. Polym. Sci..

[22] Kubo S., Kadla J.F. (2003). The formation of strong intermolecular interactions in immiscible blends of poly(vinyl alcohol) (PVA) and lignin. Biomacromolecules.

[23] Uraki Y., Nakatani A., Kubo S., Sano Y. (2001). Preparation of activated carbon fibers with large specific surface area from softwood acetic acid lignin. J. Wood Sci..

[24] Sudo K., Shimizu K. (1992). A new carbon fiber from lignin. J. Appl. Polym. Sci..

[25] Sudo K., Shimizu K., Nakashima N., Yokoyama A. (1993). A new modification method of exploded lignin for the preparation of a carbon fiber precursor. J. Appl. Polym. Sci..

[26] Chatterjee S., Clingenpeel A., McKenna A., Rios O., Johs A. (2014). Synthesis and characterization of lignin-based carbon materials with tunable microstructure. RSC Adv..

[27] Kleinhans H., Salmén L. (2016). Development of lignin carbon fibers: Evaluation of the carbonization process. J. Appl. Polym. Sci..

[28] Baumberger S., Dole P., Lapierre C. (2002). Using transgenic poplars to elucidate the relationship between the structure and the thermal properties of lignins. J. Agric. Food Chem..

[29] Brodin I., Ernstsson M., Gellerstedt G., Sjöholm E. (2012). Oxidative stabilisation of kraft lignin for carbon fibre production. Holzforschung.

[30] Bozell J.J., Black S.K., Myers M., Cahill D., Miller W.P., Park S. (2011). Solvent fractionation of renewable woody feedstocks: Organosolv generation of biorefinery process streams for the production of biobased chemicals. Biomass Bioenergy.

[31] Tao J., Hosseinaei O., Delbeck L., Kim P., Harper D.P., Bozell J.J., Rials T.G., Labbe N. (2016). Effects of organosolv fractionation time on thermal and chemical properties of lignins. RSC Adv..

[32] Sluiter A., Hames B., Ruiz R., Scarlata C., Sluiter J., Templeton D. (2008). Determination of Ash in Biomass.

[33] Sluiter A., Hames B., Ruiz R., Scarlata C., Sluiter J., Templeton D., Crocker D. (2008). Determination of Structural Carbohydrates and Lignin in Biomass.

[34] Granata A., Argyropoulos D.S. (1995). 2-Chloro-4,4,5,5-tetramethyl-1,3,2-dioxaphospholane, a reagent for the accurate determination of the uncondensed and condensed phenolic moieties in lignins. J. Agric. Food Chem..

[35] Crestini C., Argyropoulos D.S. (1997). Structural analysis of wheat straw lignin by quantitative ^31^P and 2D NMR spectroscopy. The occurrence of ester bonds and α-*O*-4 substructures. J. Agric. Food Chem..

[36] ASTM C1557-14 (2014). Standard Test Method for Tensile Strength and Young’s Modulus of Fibers.

