# Supplementary material for: Improving Processing and Performance of Pure Lignin Carbon Fibers through Hardwood and Herbaceous Lignin Blends
Source: Int J Mol Sci. 2017 Jul 1;18(7):1410. doi: 10.3390/ijms18071410 (PMC5535902; doi:10.3390/ijms18071410)
Supplement: Supplementary file 1 [file ijms-18-01410-s001.pdf]

Supplementary Materials: Improving Processing and Performance of Pure Lignin Carbon Fibers through Hardwood and Herbaceous Lignin Blends

Omid Hosseinaei, David P. Harper, Joseph J. Bozell and Timothy G. Rials

Table S1. Tukey test results for mechanical properties of carbon fibers made from different ratios of switchgrass (SG) and yellow poplar (YP) lignin blend (*w*/*w*).[a,b]

Source	Stabilization rate (°C min^{-1})	Tensile strength (MPa)	Tensile modulus (GPa)
50% YP:50% SG	0.05	BC	A
50% YP:50% SG	0.1	BCD	A
50% YP:50% SG	0.2	BCD	A
50% YP:50% SG	0.5	CD	AB
75% YP:25% SG	0.05	B	A
75% YP:25% SG	0.1	CD	CD
75% YP:25% SG	0.2	CD	CD
75% YP:25% SG	0.5	EF	D
85% YP:15% SG	0.05	A	A
85% YP:15% SG	0.1	BC	A
85% YP:15% SG	0.2	DE	BC
85% YP:15% SG	0.5	F	D

[a] Interaction of two factors (blend ratio and stabilization rate).

[b] Samples not connected by same letters are significantly different ($p < 0.01$).
